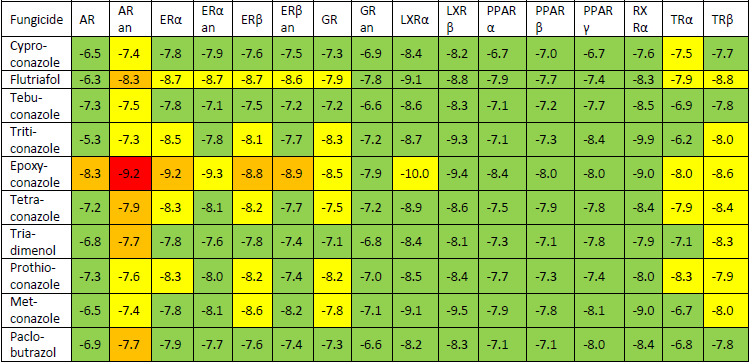# Prediction of ADME-Tox properties and toxicological endpoints of triazole fungicides used for cereals protection

**DOI:** 10.5599/admet.668

**Published:** 2019-05-21

**Authors:** Ionuţ Mădălin Gridan, Alecu Aurel Ciorsac, Adriana Isvoran

**Affiliations:** 1 Department of Biology-Chemistry and Advanced Environmental Research Laboratories, West University of Timişoara, Timişoara, Romania; 2 Department of Physical Education and Sport, University Politehnica Timişoara, Timişoara, Romania

**Keywords:** oral bioavailability, endocrine disruption, carcinogenicity, mutagenicity, cardiotoxicity

## Abstract

Within this study we have considered 9 triazole fungicides that are approved to be used in European Union for protecting cereals: cyproconazole, epoxiconazole, flutriafol, metconazole, paclobutrazole, tebuconazole, tetraconazole, triadimenol and triticonazole. We have summarized the few available data that support their effects on humans and used various computational tools to obtain a widely view concerning their possible harmful effects on humans. The results of our predictive study reflect that all triazole fungicides considered in this study reveal good oral bioavailability, are envisaged as being able to penetrate the blood brain barrier and to interact with P-glycoprotein and with hepatic cytochromes. The predictions concerning the toxicological endpoints for the investigated triazole fungicides reveal that they. reflect potential of skin sensitization, of blockage of the hERG K+ channels and of endocrine disruption, that they have not mutagenic potential and their carcinogenic potential is not clear. Epoxiconazole and triadimenol are predicted to have the highest potentials of producing numerous harmful effects on humans and their use should be avoided or limited.

## Introduction

Pesticides are a broad class of chemical compounds that are deliberately used on a planetary basis since several decades in the control of pests and vectors of diseases. In spite of the benefits of their use, their continued application and their release into various ecosystems has become a matter of concern for both humans and environment [1]. The general population is exposed to low dose of pesticides repeated over time through the food, domestic use and environment [2]. A significant risk of exposure to pesticides is registered by the people engaged in agriculture [3] and for those people living close to a workplace that uses pesticides [4]. Literature data suggest the relationship between pesticide exposure and several human pathologies: cancer, endocrine disruption, diabetes, neurologic and reproductive disorders [5,6].

There are more than 1000 active ingredients on various types of pesticides used worldwide [7]. Furthermore, the formulation of new pesticides is growing due to the appearance of resistant pests, growing global population and regulation of pesticides. The use of some pesticides such as carbamates and organophosphates decreased since 2007, but the use of triazoles have significantly increased after 2007 [8]. Triazole agents are heterocyclic compounds having a five-membered ring of two carbon and three nitrogen atoms that are widely used as antifungal agents in both medicine and in agriculture [9]. They represent around a third of the fungicides used for the protection of crop yields [10] and an important fungicide category used in the prevention of cereals diseases [11].

**Figure 1.**
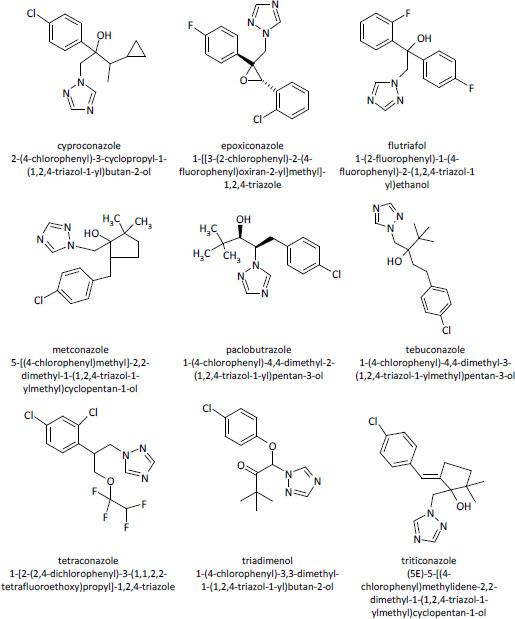
Structural formulas of triazole fungicides considered in the present study, their common and IUPAC names

The residues of triazole agricultural fungicides have been frequently detected in numerous human and environmental media and there are reported toxicity data that have led them as compounds with health concern [12]. Within this study we focus our attention on the triazole fungicides that are approved to be used in European Union for protecting cereals [13]: cyproconazole, epoxiconazole, flutriafol, metconazole, paclobutrazole, tebuconazole, tetraconazole, triadimenol and triticonazole. The structural formulas of these compounds are illustrated in the Figure 1. The routes of exposure for these chemicals usually are inhalation and dermal contact, but the oral route can be also considered as they could be found in food [4,6].

Regulatory agencies establish standard approaches to risk assessment of chemicals. Taking into account the ethics principles of conducting large-scale, long-term and highly invasive toxicity tests in humans, the risk assessment tests are usually based on toxicity studies conducted in animals. There also are important data resulting from accidental human exposures to chemicals (occupational exposure, industrial accidents, unintentional environmental releases) that are considered when setting regulatory standards to protect public health. Both scientific community and large society is divided in supporters and critics, and there are arguments for and against the intentional human dosing studies [14]. Transfer of data obtained through animal tests to humans usually depend on the ability of measuring the same endpoints in animals and data obtained from studies on humans subjects are considered more relevant for assessing toxicity than data obtained from tests on animals.

Considering the costs and the ethical concerns on using both humans and animals for testing purposes, the role of computational approaches in hazard assessment become recognized. The amount and diversity of data obtained through experimental toxicity studies allowed the building of truthful computational models for toxicology assessment. It also conducted to the development of various computational tools that can be used for hazard assessment. They are recognized by the Organization of Economic and Co-operation Development (OECD) [15] and European Food Safety Association [16] and are constantly used nowadays in assessing the toxicological effects of various chemicals on humans [17-21]. Besides the large applicability of these tools for human hazard assessment, there are some limitations mostly related to the predictability of training models and to the fact that they do not take into account the dose. We use some of these computational tools in our study and taking into account that they are meant to predict the effects on humans, for validation purposes we have only collected data obtained from human exposure on triazole fungicides.

Specific literature data contains some information concerning the effects of these triazole fungicides on the human health. We have collected data from the specific literature and toxicity databases: TOXNET/Hazardous Substances Data Bank (HSDB) [https://toxnet.nlm.nih.gov], Occupational Safety and Health Administration (OSHA) [https://www.osha.gov], National Institute of Occupational Safety and Health (NIOSH) / Pocket Guide to Chemical Hazards [http://www.aresok.org/npg/nioshdbs/npg/default.html], PAN Pesticide Database (http://www.pesticideinfo.org), PubChem (https://pubchem.ncbi.nlm.nih.gov), and Pesticides Properties DataBase (PPDB, https://sitem.herts.ac.uk/aeru/ppdb/en) available on January-February 2019. Available information is presented in Table 1.

NIOSH and OSHA databases do not contain data concerning the toxic effects of investigated triazole fungicides. Also, there is no information concerning their cardiotoxicity. Besides the information presented in table 1, there are other few known effects of triazole fungicides: flutriafol is known to produce anaemia (PPDB), paclobutrazole is considered harmful if inhaled or swallowed (PPDB), tebuconazole is considered harmful if swallowed (PubChem) and triadimenol may cause nausea, headache, sneezing and vomiting (TOXNET). Data in Table 1 illustrate that toxicological data from human exposure to pesticides are limited and sometimes controversial.

The aim of this study is to use various computational algorithms to predict the ADME-Tox profiles and toxicological endpoints of the triazole fungicides used for cereals protection in European Union.

## Method

Specific literature is abundant in computational tools available for predicting ADMET profiles and biological effects of chemicals (for more information concerning available tools, please visit http://www.vls3d.com/index.php/links/chemoinformatics/admet/admet-and-physchem-predictions-and-related-tools). We have selected for our study a few computational tools that are free accessible online, are continuously updated, are robust and their accuracy of predictions is higher than 70 %.

FAFDrugs (now arrived to version 4) is an online tool that can be used for the computational prediction of the ADMET profile based on filtering rules that take into account some physicochemical descriptors (molecular weight, polar surface area, log *P*, number of hydrogen bonds donors and acceptors, or number of rigid or rotatable bonds, etc.) [22]. It requires as input the structural data file (SDF) of the investigated compound and outputs a summary result page and a detailed result page for every analysed compound. The summary result page contains a brief statistical summary of the filtering process with graphical representations of the distributions of numerous properties, illustrating all computed values. The detailed result page contains a list of all encountered problems (if it is the case), radar plots illustrating how the compound’s properties fit into the defined physicochemical filter, a principal component analysis mapping the analysed compound into the oral chemical space of drugs, the oral bioavailability assessment considering Lipinski, Veber, Egan and Bayer rules and a drug safety profiling based on the GSK 4/400 rule, Pfizer 3/75 rule, estimation of phospholipidosis inducing and the Lilly MedChem rules with a minimum of 70 % of accuracy. When computing the Lilly MedChem rules [23], the *regular* demerit level has been applied.

SwissADME [24] computational facility has been used to predict pharmacokinetics of investigated triazole fungicides. For every investigated compound, SwissADME outputs predictions concerning the: passive human gastrointestinal absorption (GI), blood-brain barrier (BBB) permeation, skin penetration coefficient, substrate or non-substrate of the permeability glycoprotein (P-gp), interaction of molecules with five major isoforms of the human cytochromes P450 (CYP1A2, CYP2C19, CYP2C9, CYP2D6, CYP3A4) known to be involved in the metabolism of numerous endogenous and exogenous compounds [24]. The statistical performance of the classification models used by SwissADME for predicting pharmacokinetics profile is between 72 % and 94 %.

As dermal exposure to triazole fungicides is not negligible, we have used PredSkin [25] computational tool to assess the skin sensitization potential of investigated compounds. This application is based on binary QSAR models of skin sensitization potential using human and murine local lymph node assay data (LLNA) (performance ranging between 70 and 84 %) and a multiclass skin sensitization potential model based on LLNA data (performance being about 73 %). The application also outputs, for every compound, a probability map illustrating the predicted contribution to skin sensitization potential of chemical fragments [25].

Pred-hERG is a web tool based on Quantitative Structure-Activity Relationship (QSAR) models built on the curated dataset of 5,984 compounds and used to predict the hERG K+ channel blockage. Three outcomes are available when using Pred-hERG: predictions using a binary model (accuracy 80 %), predictions using a multi-class model (accuracy 70 %) and a probability map of atomic contribution to hERG K(+) channels blockage [26].

CarcinoPred-EL (Carcinogenicity Prediction using Ensemble Learning methods) is a web server allowing the classification of chemical compounds as *carcinogens* or *non-carcinogens* starting from their two-dimensional structures [27]. This web server incorporates three ensemble learning models (Ensemble XGBoost, Ensemble SVM and Ensemble RF) for predicting the carcinogenicity of chemicals. Ensemble XGBoost model gives the highest accuracy of predictions (70.1 %) [27]. Mutagenicity of considered triazole fungicides has been predicted using Ames test implemented under the open source Toxtree software [28]. This is an open source application that estimates mutagenicity by applying a decision tree approach based on Benigni and Bosa rules [29] with an accuracy of about 79 %.

Endocrine disruption potential of investigated fungicides has been evaluated using ENDOCRINE DISRUPTOME computational tool [30]. It uses the molecular docking approach based on AutoDock Vina algorithm to predict the interactions between the investigated chemicals and human nuclear receptors: androgen receptor, estrogen receptors α and β, glucocorticoid receptor, liver X receptors α and β, peroxisome proliferator activated receptors α, β/δ and γ, retinoid X receptor α and thyroid receptors α and β [24]. In the results section, three thresholds, calculated from the docking score and the validation experiments and expressed as sensitivity (SE) parameter, were set for every structure allowing a division into 4 probability binding classes: compounds with high probability of binding (SE<0.25), compounds with intermediate probability of binding (0.25<SE<0.50), compounds with moderate probability of binding, (0.50<SE< 0.75) and Compounds with low probability of binding (SE>0.75) [30].

## Results and Discussion

ADMET profiles of investigated fungicides have been obtained using FAFDrugs4 and respectively SwissADME computational facilities and are illustrated in tables 2 and 3. Data presented in Tables 2 and 3 illustrate that all investigated triazole fungicides have good oral bioavailability, can be easily absorbed in the gastrointestinal tract and, consequently, are able to reach the systemic blood circulation and to produce various biologic effects.

Every investigated compound is predicted to have at least a mean toxicity when applying Pfizer rule, the toxicity being related to their content in halogen atoms and, in the case of epoxiconazole, the epoxide group also reflect toxicity. Epoxiconazole and triadimenol also do not pass Lilly MedChem rules meaning that these two pesticides may illustrate activities that damage proteins [23]. None of investigated compounds is predicted to induce phospholipidoses (data not shown).

All investigated fungicides are predicted as being able to penetrate the blood brain barrier and it illustrates their potential to affect the central nervous system, and/or the transport of nutrients, drugs and waste products into and out of the brain. Available information illustrates that most of investigated triazoled fungicides do not produce human neurotoxicity (see Table 1), but there are published data exemplifying neurotoxic effects of these compounds on rats [31-32]. Epoxyconazole and triticonazole are predicted as P-gp substrates and it illustrates their possible active efflux both from the gastrointestinal tract to the lumen and from the brain, being known that one of the roles of P-gp is to protect the central nervous system from xenobiotics. The predictions are in good agreement with literature data revealing that the triazoles are substrates and/or inhibitors of transport proteins in the ATP-binding cassette transporter protein family [33]. Furthers studies are necessary for assessing the risk of neurotoxicity of trizole fungicides on humans.

All investigated fungicides are predicted to be able to inhibit CYP2C19 and some of them are also able to inhibit CYP1A2, CYP2C9 and/or CYP2D6. These predictions are in good agreement with published data concerning the interactions of triazole antifungal human drugs with hepatic CYPs. Literature data reveal that triazole antifungal agents as human drugs are substrates and inhibitors of various CYPs [34], including CYP3A4, CYP2C19 and CYP2C9 that are involved in catalysing the triazole biotransformation [35]. It is possible that investigated triazole fungicides to be metabolized by CYPs. In this case, the polymorphisms within these enzymes may conduct in their accumulation into the human organism. The inhibition of human CYPs involved in the metabolism of many endogenous compounds and of xenobiotics by the investigated triazole fungicides may conduct to pharmacokinetics-related drug-xenobiotics interactions leading to toxic and/or adverse effects. It underlines the importance of predictions of which isoforms of CYPs are affected by triazole fungicides.

**Figure 2.**
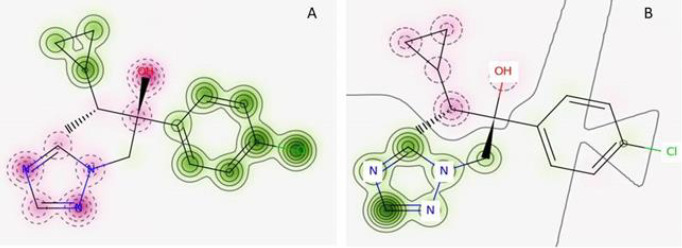
The redicted probability map of the predicted contribution of chemical fragments of cyproconazole to: **(A)** skin sensitization potential; **(B)** blockage of the hERG K+ channels. Green color corresponds to atoms illustrating an increase in skin sensitization **(A)** or towards blockage of hERG **(B)**, pink color corresponds to atoms emphasizing a decrease in skin sensitization **(A)** or in hERG blockage **(B)** and gray lines delimit the region of split between the positive and negative contributors toward skin sensitization **(A)** or hERG blockage **(B)**. More continuous contour lines mean an increased pozitive contribution and more dashed contour lines mean an increased negative contribution of an atom to skin sensitization **(A)** or hERG blockage **(B)** respectively.

The predicted values of the skin penetration coefficients in logarithmic values (log *K*_p_) of investigated triazole fungicides were compared with those of diclofenac (a compound with a good skin penetration, log *K*p=-4.96), and of oubain (a compound that is not able to penetrate skin, log *K*_p_ = -11.07) [22]. The more negative value of computed log *K*_p_ means lower skin permeability for the investigated compound. This comparison illustrates the medium ability of triazole fungicides to penetrate skin and it is in good correlation with data proving that some of the investigated fungicides produce skin irritations (see Table 1). We have also evaluated the skin sensitization potential of investigated triazole fungicides using Pred-Skin computational tool and the results are illustrated in Table 4. The probability map illustrating the predicted contribution to skin sensitization potential of chemical fragments of cyproconazole when using the binary prediction based on human skin sensitization model is shown in Figure 2 A.

This picture illustrates the positive contribution to skin sensitization of the chlorophenyl and cyclopropyl groups, but not of triazole ring. Table 4 illustrates that all investigated triazole fungicides are predicted as skin sensitizers. Available information concerning the ability of some of the investigated fungicides to produce skin irritations corresponds to an acute exposure. Our predictions reveal that exposure (especially when prolonged) to these compounds may conduct to skin sensitization, a complex immunological disease that have an important impact on quality of life and on working ability.

Table 5 contains predictions of the following toxicological endpoints of the investigated triazole fungicides: cardiotoxicity (blocking potential of h-ERG K+ channels) obtained using Pred-hERG tool, mutagenicity obtained using Toxtree software and carcinogenicity using both Toxtree and CarcinoPred-El computational facilities. The two models used by Pred-hERG tool give contradictory predictions. For all the investigated tiazole fungicides, the use of multiclass model do not predict the blockage of the hERG K+ channels potential, but the use of the binary model reveal their potential to block the hERG K+ channels. Taking into account that the binary model has a higher accuracy of predictions, we consider that investigated triazole fungicides reflect potential to block the hERG K+ channels. Figure 2B shows the probability map of the predicted contribution of atoms and/or fragments of cyproconazole toward blockage of the hERG K+ channels. It illustrates that triazole fragment and the halogen atom reveal potential of the blockage of the hERG K+ channels. Besides the triazole ring, there is at list one fragment containing a halogen atom attached to the phenyl group in every of investigated fungicides (see Figure 1) and it reflects the hERG K+ channel-blocking potential of these pesticides. Our findings are in good correlation with published data revealing that compounds containing a polar group (such as a halogen atom) attached to the phenyl ring at one end of the molecule reveal hERG K+ channel-blocking potential [36,37]. Furthermore, the class of antifungal triazoles used as human drugs [38] and other compounds containing the triazole ring [39] are recognized as being able to cause cardiac dysrhythmias by blocking hERG K+ channels in the heart.

The outcomes of CarcinoPred-El computational tool reveal carcinogenic potential for flutriafol, paclobutrazole, triadimenol and triticonazole when using XGBoost ensemble learning model, this model giving the highest accuracy of predictions. Only flutriafol is predicted as carcinogen by all models considered by CarcinoPred-EL tool. Toxtree outcomes are quite different by comparison with those of CracinoPred-EL and reveal both genotoxic and non-genotoxic carcinogenicity for epoxiconazole and, excepting flutriafol, non-genotoxic carcinogenicity for all the other investigated triazole fungicides. Literature data show that most of the investigated triazole fungicides are considered as probable human carcinogen. Usually, the predictions obtained with the two computational tools are not in agreement each other or with known information concerning the carcinogenic effects of triazole fungicides. It underlines the need of further investigations concerning the carcinogenic potential of triazole fungicides on humans.

Predictions obtained using ENDOCRINE DISRUPTOME computational tool reflected that investigated triazole fungicides are able to influence the activity of some of the human nuclear receptors, as presented in Table 6. Red boxes correspond to the high probability of binding, orange boxes to intermediate probability of binding, yellow boxes to moderate probability of binding and green boxes correspond to low probability of binding of triazole fungicides to these receptors. All investigated triazole fungicides are predicted as revealing antagonistic effects on the androgen receptor. Epoxiconazole is the compound predicted as reflecting the highest endocrine disruption potential as it interacts with numerous human nuclear receptors. The predictions of ENDOCRINE DISRUPTOME tool reveal that the most affected nuclear receptors by the analysed triazole fungicides are: AR, ERα, ERβ, GR, TRα and TRβ. This result is in good agreement with published data revealing the ability of triazole fungicides to interact with nuclear receptors, especially with the androgen and estrogen receptors [12, 40-33].

## Conclusions

Within this study we have considered 9 triazole fungicides commonly used for cereal crops protection. We have summarized the available data that support their effects on humans and we have also used various computational tools to predict their harmful effects. Available data are incomplete and the outcomes of this predictive study are meant to obtain a widely view concerning the harmful effects of these pesticides. The predictions that we have obtained using various tools usually are in good agreement with each other and with available data, this accord increasing their relevance. Triazole fungicides considered in this study are predicted as revealing high oral bioavailability, being able to penetrate the blood brain barrier, and to interact with P-glycoprotein and with hepatic CYPs.

The predicted toxicological endpoints of studied triazole fungicides are: (i) skin sensitising potential; (ii) blockage of the hERG K+ channels and (iii) endocrine disruption potential, the most affected human nuclear receptor being the androgen receptor, estrogen receptors α and β, glucocorticoid receptor and thyroid receptors α and β. Triazole fungicides considered in this study are predicted to have not mutagenic potential and their carcinogenic potential is not clear.

Of the investigated fungicides, our predictions show that epoxiconazole and triadimenol seem to have the highest potentials of producing harmful effects on humans and their use should be avoid or limited. As the use of triazole fungicides is increasing, the outcomes of our study are important for both population and professional exposure.

## Figures and Tables

**Table 1.** Known human health effects of triazole fungicides used for cereals protection

Triazole fungicide	Skin/eye irritations	Respiratory tract irritant	Carcinogenicity/Mutagenicity	Endocrine disruption potential	Reproductive toxicity	Hepato-toxicity	Neuro-toxicity
cyproconazole	Mild eye irritant (TOXNET), skin and eye irritant (PPDB)	Yes (PPDB)	Probable carcinogen (TOXNET, PAN) /No data found.	Inhibition of aromatase activity, decrease of estrogens production (PPDB)	No data found.	Possible liver toxicant (PPDB)	No (PPDB)
epoxiconazole	No (PPDB)	No (PPDB)	probable carcinogen (PPDB, PubChem, PAN)/ No data found.	inhibition of aromatase activity, decrease of estrogen production (PPDB), suspect to produce endocrine disruption (PAN)	No data found.	Liver toxicant (PPDB)	No (PPDB)
flutriafol	NO (PPDB)	Yes (PPDB)	No (PPDB, PAN)/ No data found.	Weak estrogen inhibition (PPDB), suspect to produce endocrine disruption (PAN)	No data found.	Possible liver toxicant (PPDB)	No (PPDB)
metconazole	NO (PPDB) Yes (PubChem)	Yes (PPDB)	No (PPDB, PAN)/ No data found.	No data found.	Suspect of damaging fertility or the unborn child. (PubChem)	Possible liver toxicant (PPDB)	No (PPDB)
paclobutrazole	Yes (PPDB, PubChem)	No data found.	NO (PPDB)/ No data found.	No data found.	Suspect of damaging fertility or the unborn child. (PubChem)	No data found.	No (PPDB)
tebuconazole	Eye irritant (PPDB)	No (PPDB)	probable carcinogen (TOXNET, PAN)/ NO (PPDB)	NO (PPDB), suspect to produce endocrine disruption (PAN)	Yes (PPDB, PubChem)	No data found.	No (PPDB)
tetraconazole	Eye irritant (TOXNET), no effects (PPDB)	No (ToxNET, PPDB)	probable carcinogen (TOXNET, PPDB, PAN)/ No (PPDB)	No (PPDB)	No data found.	Liver toxicant (PPDB)	No (PPDB)
triadimenol	Yes (TOXNET, PPDB)	YES (TOXNET, PPDB)	probable carcinogen (PPDB, PAN)/ No data found.	Yes (TOXNET), estrogenic effect (PPDB), suspect to produce endocrine disruption (PAN)	Yes (PPDB, PubChem)	Liver toxicant (PPDB)	No data found.
triticonazole	No (PPDB)	No (PPDB)	probable carcinogen (TOXNET), noncarcinogen (PPDB)/ No data found.	No data found.	No data found.	No data found.	No data found.

**Table 2.** Prediction of the ADMET profiles obtained using the FAFDrugs4 computational tool for the triazole fungicides considered in this study: green boxes correspond to rules that are respected, orange boxes correspond to rules that are partially violated and red grey boxes correspond to rules that are not respected.

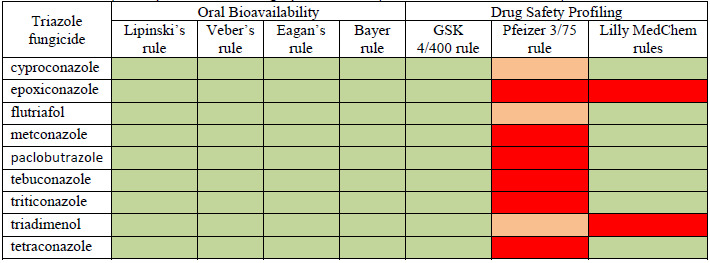

**Table 3.** Prediction of the pharmacokinetics profiles of the triazole fungicides using SwissADME computational tool: GI- gastrointestinal absorption, BBB- blood brain barrier permeant, P-gp – P-glycoprotein, CYP – human cytochrome, log *K*_p_ - skin penetration coefficient in logarithmic scale.

Active substance	GI absorption	BBB permeant	P-gp substrate	CYP1A2 Inhibitor	CYP2C19 Inhibitor	CYP2C9 Inhibitor	CYP2D6 Inhibitor	CYP3A4 Inhibitor	log *K*_p_
cyproconazole	High	Yes	No	No	Yes	No	No	No	-6.02
epoxiconazole	High	Yes	Yes	No	Yes	Yes	Yes	No	-5.87
flutriafol	High	Yes	No	No	Yes	No	Yes	No	-6.50
metconazole	High	Yes	No	No	Yes	No	Yes	No	-5.52
paclobutrazole	High	Yes	No	No	Yes	No	No	No	-5.82
tebuconazole	High	Yes	No	Yes	Yes	No	Yes	No	-5.55
triticonazole	High	Yes	Yes	No	Yes	Yes	No	No	-5.87
triadimenol	High	Yes	No	No	Yes	No	No	No	-5.92
tetraconazole	High	Yes	No	Yes	Yes	Yes	No	No	-6.04

**Table 4.** Prediction of skin sensitization potential of the considered triazole fungicides: LLNA - murine local lymph node assay, DRPA - direct peptide reactivity assay, h-CLAT - human cell line activation test. Green boxes correspond to predictions of non-sensitizer potential, orange boxes to predictions of moderate skin sensitizer potential and red boxes illustrate predictions of skin sensitizer potential. Numbers in parenthesis correspond to the accuracy of every prediction.

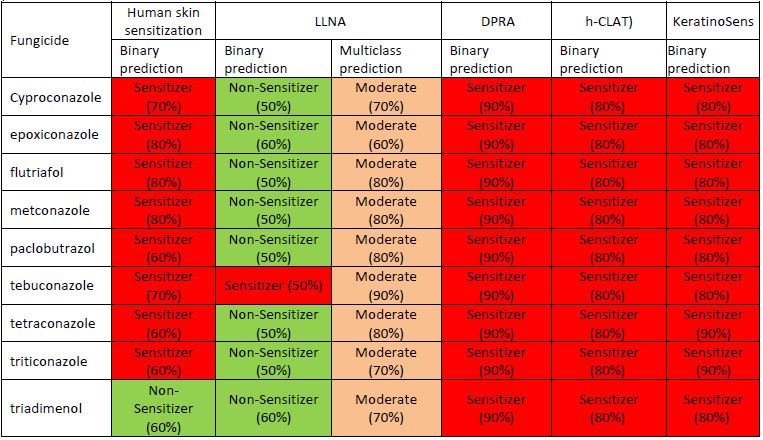

**Table 5.** Predictions concerning the following toxicological endpoints: cardiotoxicity, carcinogenicity and mutagenicity of investigated triazole fungicides.

Fungicide	h_ERG channel blockage predictions		CarcinoPred-EL predictions for carcinogenicity			Toxtree predictions		
	binary model	multiclass model	XGBoost model	RF model	SVM model	genotoxic carcino-genicity	non genotoxic carcino-genicity	Ames muta-genicity
cyproconazole	blocker (70 %)	Non-Blocker (70 %)	No	No	No	No	Yes	No
epoxiconazole	blocker (70 %)	Non-Blocker (60 %)	No	No	No	yes	Yes	No
flutriafol	blocker (60 %)	Non-Blocker (70 %)	Yes	Yes	Yes	No	No	No
metconazole	blocker (80 %)	Non-Blocker (70 %)	No	No	No	No	Yes	No
paclobutrazole	blocker (70 %)	Non-Blocker (60 %)	Yes	No	No	No	Yes	No
tebuconazole	blocker (70 %)	Non-Blocker (60 %)	No	No	No	No	Yes	No
tetraconazole	blocker (70 %)	Non-Blocker (70 %)	No	No	No	No	No	No
triadimenol	blocker (60 %)	Non-Blocker (70 %)	Yes	No	No	No	Yes	No
triticonazole	blocker (70 %)	Non-Blocker (60 %)	Yes	No	No	No	Yes	No

**Table 6.** Prediction of the endocrine disruption potential of investigated triazole fungicides: red boxes correspond to the high probability of binding, orange boxes correspond to intermediate probability of binding, yellow boxes correspond to moderate probability of binding and green boxes correspond to low probability of binding of triazole fungicides to these receptors.